# The perception of the neighborhood environment changes after participation in a pedometer based community intervention

**DOI:** 10.1186/1479-5868-9-33

**Published:** 2012-03-27

**Authors:** Birgit Wallmann, Heleen Spittaels, Ilse De Bourdeaudhuij, Ingo Froboese

**Affiliations:** 1 Centre of Health, German Sport University Cologne, Cologne, Germany; 2 Institute of health promotion and clinical movement science, German Sport University Cologne, Cologne, Germany; 3 Faculty of Medicine and Health Sciences, Department of Movement and Sports Sciences, Ghent University, Ghent, Belgium

**Keywords:** Physical activity, 3000 steps more per day, Health behaviour, Environmental questionnaire

## Abstract

**Background:**

The aim of this study was to investigate whether the perception of the neighbourhood environment alters when changing the physical activity behaviour through a pedometer intervention.

**Findings:**

The intervention was implemented for 15 weeks in a small village in Germany, and was based on the individual baseline activity level. Eighty-two inhabitants participated in the study and completed an environmental questionnaire before and after the intervention. Results showed that after the intervention the participants perceived a lower distance to local facilities, a higher availability of bike lanes and infrastructures, a better maintenance of infrastructure, a better network and a safer traffic situation.

**Conclusion:**

This suggests that a change in the levels of physical activity merges the levels of exposure to the environment which results in different environmental perceptions.

## Introduction

There is a growing body of research showing the impact of the environment on physical activity (PA) [1-3]. Studies, using objective as well as subjective measures, indicate that mixed land use, residential density, access and quality of sidewalks, transportation system, recreational resources and aesthetics can affect PA behaviour [4]. Literature results remain uncertain concerning the question whether objective (e.g. GIS) or subjective measures (perceptions) are more important to describe the relationship between the environment and PA [5,6]. Till now research drew a one-directional view on how the perception of the environment influences PA behaviour, although it also could be conceivable that the perception is influenced by PA. Only one study examined changes in environmental perceptions over time and associations between changes in perceptions and PA, nevertheless only the perceived home and facility environments were included [7]. Thus it is of interest to study how alterable the individual perception of the environment is by just changing the PA behaviour. The aim of this study was to investigate whether the perception of the neighbourhood environment alters when changing the PA behaviour through a "3000 steps more per day" intervention.

## Methods

### Study design

The intervention "3000 steps more per day" was conducted as a three month pre-post intervention study with baseline data collection in January/February 2010 and follow-up data in May 2010 in a community setting. The intervention community was a small German rural village called Berghausen approximately 50 km east of Cologne with 1237 inhabitants (condition from 31.12.2007). Residents were invited to an information evening on the topic of "Physical Activity and Health" and were introduced afterwards to the intervention. Interested inhabitants were asked to sign up for the intervention and requested to buy the suggested pedometer. To identify the baseline activity level, all participants wore an individual set up pedometer (stride length, body weight) with invisible taped screen for seven consecutive days. After seven days the data storage of the pedometer was read out and the individual mean daily step activity was calculated. The participants were urged to accumulate additional 3000 steps per day for the duration of 15 weeks and wore the pedometer throughout the intervention with a now visible screen. During the intervention phase, the pedometer data storage was read out every month (four times in total) and walking behaviour during the intervention was calculated as a mean of all intervention days. Pre- and post measures included questionnaires concerning PA related environmental factors.

### Sample

About 160 people attended the information evening. From these, 123 inhabitants (38 male/74 female/1 not specified) signed up for the intervention (approx. 77% response rate) which represented 9.9% of the whole village population. At the beginning of the intervention 21 participants were withdrawn from the analysis because they did not buy the suggested pedometer, which made step data analysis impossible. Eight participants dropped-out during the intervention in terms of missing pedometer data (7.8%). Due to missing questionnaires (n = 9) and an age younger than 16 years (n = 3), 82 inhabitants (30 male/52 female), all German nationality, were included in the analysis. Mean age of the sample was 52.8 ± 13.0 years and mean body mass index was 27.3 ± 4.5 kg/m^2^. Based on the German school system 35.4% (n = 29) had a lower education (≤ 10 school years) and 64.6% (n = 53) had a higher school education of more than ten school years. All participants gave their written informed consent to participate.

### "3000 steps more per day" intervention

The intervention "3000 steps more per day" was suggested because it has been shown that the accepted PA recommendation of 30 minutes of moderate intense PA [8] can be translated into 3000 steps [9,10] and therefore be an adequate PA target. Compared to other pedometer interventions with a benchmark of i.e.10.000 steps per day [11,12] the used intervention target takes the individual baseline PA more into account. The administered intervention did not aim to change the perception of the environment, nor the actual environment.

During the intervention, PA was promoted in the village through a wide range of optional PA events. Nine regular activities were offered weekly (i.e. morning walks, dog walking, nordic walking, active walking, gymnastics, fit & active program for seniors) and 23 singular events especially during the weekend were carried out (i.e. guided walking tours, different theme nights on health topics, a historical village tour, geocaching, walking and collecting garbage, a soccer tournament, a carnival procession). The optional program was provided by the local sports club, the local beautification club, the auxiliary fire brigade as well as individual villagers. However, it was highlighted to the participants that the PA program was only optional and not obligatory during the intervention.

### Measures

#### Physical activity

Objective PA data were collected using the OMRON HJ-720ITC. The HJ-720ITC pedometer features dual piezoelectric sensors, allowing accurate step counting to occur when worn in both vertical and horizontal positions. The HJ-720ITC offers PC downloading capabilities, including a 7-day recall on the pedometer display and a 41-day storable memory for measures of daily step counts, aerobic step counts, prediction of caloric expenditure, and distance walked. For the HJ-720ITC a good accuracy (all absolute percent errors < 3%) and a good reliability (coefficient of variation value < 2.1%) for step activity has been reported [13].

#### Measures of perceived environment

The perceived environment was assessed self-administered by the German version of the European Environmental Questionnaire ALPHA. This questionnaire consisted of nine themes of the neighbourhood (types of residences, distances to local facilities (both five point scale), walking or cycle infrastructure, maintenance of infrastructure, neighbourhood safety, pleasure and aesthetics of the neighbourhood, cycling and walking network (all four point scale), home environment, workplace or study environment (both dichotomized "yes, "no")) with a total of 49 items and has been introduced elsewhere [14,15]. The reliability of the instrument has been shown (ICC 0.71-0.87) and it was translated from English into German followed by cognitive testing [15]. For the German context validity and reliability has been confirmed [16].

Background variables like gender, age, objectively measured anthropometric variables (body mass index), education and nationality were additionally assessed.

### Data analysis

We used the data processing software PASW^© ^(Version 18) for all statistical analyses. To distinguish between participants who were able to achieve the intervention goal of "3000 steps more per day" throughout the intervention ("achievers"), and the participants who were not able to perform the intervention goal ("non-achievers"), the difference between the mean steps during the 15 week intervention phase and the mean steps during the 7-day baseline phase was calculated.

Distribution of the environmental sum scores approached normality. Repeated measures ANOVAs were executed with the different environmental perception items as the within subject factors. "Achievers" vs. "non-achievers" were included as the first between subject factors. To investigate the influence of baseline PA levels, we also split the group into two on the basis of the median of the baseline PA (< 5549 steps (n = 41)/> 5549 steps (n = 41)), and included this as the second between subject factor. Statistical significance was set at a level of .05.

## Results

### PA measures and achievement of intervention goal "3000 steps more per day"

Mean number of steps increased from 5977 (± 2327) steps per day at baseline to 9091 (± 3007) steps per day during the intervention. For females the difference between intervention and baseline accounted 3072 (± 2012), for males 3186 (± 2063) steps per day. Throughout the 15 week intervention period, 44 participants (53.7%) were able to achieve the aim of "3000 steps more per day" and are further described as "achievers". 38 participants (46.3%) achieved less than "3000 steps more per day" and are referred to as "non-achievers".

### Adhesion in optional PA events

69 participants joined at least one optional regular or singular event during the intervention. For singular events all in all 45 different participants joined at least one offer with a mean of 1.9 (± 1.2) events. For regular events, 51 participants adhered at least one session of one event. On average they visited 10.0 (± 8.7) sessions during the intervention.

### Changes in perception after intervention

Table 1 shows the changes in environmental perceptions after the intervention. After the intervention participants perceived a lower distance to local facilities (F = 7.20; *p *< 0.01) and a higher availability of infrastructures (F = 6.12; *p *< 0.05), so the environment was perceived as more walkable after the intervention. The perception also increased for the availability of bike lanes, but only for participants who were more active at baseline (T = -3.86; *p *< 0.001). Another favouring result was a better perception of the maintenance of infrastructures. However in participants with low baseline PA, only "achievers" increased their perception (T = -2.612; *p *< 0.05) and for participants with high baseline PA, only the "non-achievers" increased their perception (T = -4.736; *p *< 0.001) after the intervention. Moreover, "achievers" perceived a safer traffic situation. Another increase was found for the perception of "network", but only for participants with low baseline PA (T = -2.45; *p *< 0.05).

**Table 1:** Results of repeated measures for changes in perception of the neighbourhood environment stratified by "achievers" (n = 44) and "non-achievers" (n = 38) (Total n = 82)

Themes of neighbour-hood		Baseline	Follow-up	Time effect	Time × achiever	Time × baseline	Time × achiever × baseline
		**Mean (SD)**	**Mean (SD)**	**F (p)**	**F (p)**	**F (p)**	**F (p)**

**Density score**	Achievers	103.0 (45.9)	97.8 (29.7)				
	
	Non-achievers	98.7 (37.6)	100.9 (37.5)				
	
	Total	110.0 (42.1)	99.2 (33.4)	0.15 (ns)	0.87 (ns)	0.09 (ns)	0.15 (ns)

**Distance score**	Achievers	22.8 (5.0)	22.5 (4.4)				
	
	Non-achievers	21.8 (4.2)	19.6 (3.9)				
	
	Total	22.3 (4.7)	21.1 (4.4)	7.20 (**)	3.39 (ns)	1.65 (ns)	0.09 (ns)

**Availability of sidewalks**	Achievers	4.4 (1.5)	4.8 (1.6)				
	
	Non-achievers	4.1 (1.4)	4.1 (1.4)				
	
	Total	4.3 (1.5)	4.5 (1.6)	1.71 (ns)	1.65 (ns)	0.09 (ns)	0.01 (ns)

**Availability of bike lanes**	Achievers	2.1 (0.5)	2.4 (0.9)				
	
	Non-achievers	2.2 (0.6)	2.5 (0.8)				
	
	Total	2.2 (0.6)	2.4 (0.8)	7.80 (**)	0.00 (ns)	5.68 (*)a	0.33 (ns)

**Availability of infrastructure**	Achievers	6.5 (1.6)	7.2 (1.9)				
	
	Non-achievers	6.3 (1.7)	6.6 (1.9)				
	
	Total	6.5 (1.7)	6.9 (1.9)	6.12 (*)	0.89 (ns)	2.08 (ns)	0.13 (ns)

**Maintenance of infrastructure**	Achievers	5.8 (1.3)	6.5 (1.8)				
	
	Non-achievers	6.3 (1.7)	7.3 (2.2)				
	
	Total	6.0 (1.5)	6.9 (2.0)	20.96(***)	0.45 (ns)	0.60 (ns)	7.56 (**)b

**Total safety**	Achievers	21.2 (2.5)	21.4 (2.5)				
	
	Non-achievers	21.7 (2.2)	21.2 (2.9)				
	
	Total	21.5 (2.3)	21.3 (2.7)	0.32 (ns)	1.63 (ns)	2.88 (ns)	1.00 (ns)

**Safety crime**	Achievers	11.1 (1.1)	10.9 (1.2)				
	
	Non-achievers	11.1 (1.1)	10.9 (1.3)				
	
	Total	11.1 (1.1)	10.9 (1.2)	1.19 (ns)	0.01 (ns)	1.11 (ns)	2.83 (ns)

**Safety traffic**	Achievers	10.1 (1.9)	10.5 (1.7)				
	
	Non-achievers	10.7 (1.3)	10.3 (2.1)				
	
	Total	10.4 (1.6)	10.4 (1.9)	0.00 (ns)	4.05 (*)	3.12 (ns)	0.05 (ns)

**Pleasure**	Achievers	12.8 (1.7)	13.1 (1.7)				
	
	Non-achievers	13.9 (1.7)	13.9 (3.5)				
	
	Total	13.3 (1.8)	13.4 (2.7)	0.08 (ns)	0.10 (ns)	2.66 (ns)	0.57 (ns)

**Aesthetics**	Achievers	9.7 (1.4)	9.9 (1.5)				
	
	Non-achievers	10.5 (1.3)	10.8 (3.4)				
	
	Total	10.1 (1.4)	10.3 (2.6)	0.75 (ns)	0.07 (ns)	2.72 (ns)	0.55 (ns)

**Network**	Achievers	7.7 (1.9)	7.9 (1.9)				
	
	Non-achievers	8.1 (2.0)	8.7 (2.1)				
	
	Total	7.9 (2.0)	8.2 (2.0)	2.93 (ns)	0.49 (ns)	5.44 (*)c	1.25 (ns)

**Connectivity**	Achievers	6.2 (1.8)	6.2 (1.7)				
	
	Non-achievers	6.6 (1.8)	6.8 (1.7)				
	
	Total	6.4 (1.8)	6.5 (1.7)	0.45 (ns)	0.40 (ns)	3.78 (ns)	1.06 (ns)

**Home**	Achievers	3.9 (1.5)	3.5 (1.1)				
	
	Non-achievers	4.0 (1.1)	3.8 (1.1)				
	
	Total	3.9 (1.3)	3.6 (1.1)	3.68 (ns)	0.97 (ns)	2.31 (ns)	1.91 (ns)

**Work/Study**	Achievers	4.2 (2.8)	3.7 (1.7)				
	
	Non-achievers	4.4 (3.1)	3.7 (1.6)				
	
	Total	4.3 (2.9)	3.7 (1.6)	1.83 (ns)	0.20 (ns)	0.33 (ns)	2.74 (ns)

*p ≤ 0.05; **p ≤ 0.01; ***p ≤ 0.001; ns = not significant (p ≥ 0.05)

a The perceptions concerning the availability of bike lanes increased in participants with high baseline PA (p < 0.001) whereas the perceptions of the participants with low baseline PA didn't change

b Maintenance of infrastructure was perceived more positively after the intervention by participants with low baseline PA, but only in the achievers" (p < 0.05); while for participants with high baseline PA, only the "non-achievers" increased their perception (p < 0.001) after the intervention

c The perception of the network increased in participants with low baseline PA (p < 0.05) whereas the perception of the participants with high baseline PA didn't change

## Discussion

The main finding of this study was that the perception of the environment altered after the walking intervention in the themes of "distance", "availability of bike lane", "availability of infrastructure", "maintenance of infrastructure", "network" and "safety traffic" without changing the environment. However there were some interactions between baseline PA and the achievement of "3000 steps more per day" in the themes of "availability of bike lane", "maintenance of infrastructure", "network" and "safety traffic" which indicates that baseline PA as well as the increase in steps (PA) has an influence on some changes in perception of the environment. It can be assumed that simply wearing the pedometer and getting aware of the personal PA goals, can lead to a higher sensitivity for the own environment. In line with the statement of Ries and colleagues [7], increasing exposure to the neighborhood environment may result in changes in environmental perceptions related to PA. Importantly, all changes in environmental perceptions were in a favorable way, considering the environment as more inviting to be physically active.

However, our results do not allow us to conclude about the direction of cause and effect, why the environmental perception changed. Either increased PA levels resulted in a higher exposure to the environment or the change in perception "caused" an increase in PA. Adding to the discussion whether objective or perceived environmental measures are necessary to understand the relationship concerning PA [2,5], our results suggest to combine both measures in future studies as the perception seems to be influenced by the exposure to the environment. This aspect should be taken into account and further research on this issue is warranted.

The strength in the present study is that the intervention was carried out in a small isolated and controlled community so that caused changes could be rather explained through the PA intervention itself and not through potential changes in the environment. However, also external influences on the environmental perception such as the season can be an issue in allowance of starting the intervention in winter and ending in spring, or even the attendance in the optional PA events. Furthermore PA was monitored during the whole intervention and not only around post-measurements which is beneficial. Nevertheless there are some limitations to the study. Firstly there was no control group and the sample size was small. Secondly a generalisation of the results is limited, because the study was just conducted in one small rural village and results could differ for urban or suburban communities. Even so, we see important findings in the results of our study which have not been investigated sufficiently in the present literature.

## Conclusion

The results of this study suggest that the perception of the environment is alterable in a favourable way by increasing PA levels, although a definitive rationale for the change cannot be given. However, these results also show that we have to be cautious by concluding from cross-sectional studies that a less favourable perceived environment is responsible for a lower PA level, since this study shows that a lower exposure to the environment seems to be related to a less favourable perception of the environment. Eventually this advises the simultaneously use of subjective and objective measures to better understand the relationship between the environment and PA.

## Abbreviations

PA: Physical activity.

## Competing interests

The authors declare that they have no competing interests.

## Authors' contributions

BW participated in the conception and the design of the present study and carried out the data acquisition, performed statistical analyses, interpreted the data, wrote and drafted the manuscript. HS and IsB contributed to the analyses and interpretation of data and provided critical revision of the manuscript. IF participated in the conception and design of the study and provided critical revision of the manuscript. All authors read and approved the final manuscript.

## References

[B1] Sallis JF Bowles HR Bauman A Ainsworth BE Bull FC Craig CL Sjostrom M De Bourdeaudhuij I Lefevre J Matsudo V Neighborhood environments and physical activity among adults in 11 countries Am J Prev Med 2009 36 484 490 10.1016/j.amepre.2009.01.031 19460656

[B2] McGinn AP Evenson KR Herring AH Huston SL Rodriguez DA Exploring associations between physical activity and perceived and objective measures of the built environment J Urban Health 2007 84 162 184 10.1007/s11524-006-9136-4 17273926 PMC2231636

[B3] Duncan MJ Spence JC Mummery WK Perceived environment and physical activity: a meta-analysis of selected environmental characteristics Int J Behav Nutr Phys Act 2005 2 11 10.1186/1479-5868-2-11 16138933 PMC1236952

[B4] Sallis JF Cervero RB Ascher W Henderson KA Kraft MK Kerr J An ecological approach to creating active living communities Annu Rev Public Health 2006 27 297 322 10.1146/annurev.publhealth.27.021405.102100 16533119

[B5] Brownson RC Hoehner CM Day K Forsyth A Sallis JF Measuring the built environment for physical activity: state of the science Am J Prev Med 2009 36 S99 S123.e112 10.1016/j.amepre.2009.01.005 19285216 PMC2844244

[B6] McCormack G Giles-Corti B Lange A Smith T Martin K Pikora TJ An update of recent evidence of the relationship between objective and self-report measures of the physical environment and physical activity behaviours J Sci Med Sport 2004 7 81 92 15214606 10.1016/s1440-2440(04)80282-2

[B7] Ries AV Dunsiger S Marcus BH Physical activity interventions and changes in perceived home and facility environments Prev Med 2009 49 515 517 10.1016/j.ypmed.2009.10.009 19853621 PMC2805038

[B8] Haskell WL Lee IM Pate RR Powell KE Blair SN Franklin BA Macera CA Heath GW Thompson PDAB Physical Activity and Public Health: Updated Recommendation for Adults From the American College of Sports Medicine and the American Heart Association Circulation 2007 116 1081 1093 17671237 10.1161/CIRCULATIONAHA.107.185649

[B9] Marshall SJ Levy SS Tudor-Locke CE Kolkhorst FW Wooten KM Ji M Macera CA Ainsworth BE Translating physical activity recommendations into a pedometer-based step goal: 3000 steps in 30 minutes Am J Prev Med 2009 36 410 415 10.1016/j.amepre.2009.01.021 19362695

[B10] Tudor-Locke C Hatano Y Pangrazi RP Kang M Revisiting "How Many Steps Are Enough?" Med Sci Sports & Exercise 2008 40 S537 S543 10.1249/MSS.0b013e31817c7133 18562971

[B11] De Cocker KA De Bourdeaudhuij IM Brown WJ Cardon GM Effects of "10,000 Steps Ghent" Am J Preventive Med 2007 33 455 463 10.1016/j.amepre.2007.07.037 18022061

[B12] Schneider PL Bassett DR Jr Thompson DL Pronk NP Bielak KM Effects of a 10,000 steps per day goal in overweight adults Am J Health Promot 2006 21 85 89 17152246 10.4278/0890-1171-21.2.85

[B13] Holbrook EA Barreira TV Kang M Validity and reliability of Omron pedometers for prescribed and self-paced walking Med Sci Sports Exerc 2009 41 670 674 10.1249/MSS.0b013e3181886095 19204582

[B14] Spittaels H Foster C Oppert J-M Rutter H Oja P Sjöström M De Bourdeaudhuij I Assessment of environmental correlates of physical activity: development of a European questionnaire Int J Behav Nutr Phys Act 2009 6 39 10.1186/1479-5868-6-39 19580645 PMC2713198

[B15] Spittaels H Verloigne M Gidlow C Gloanec J Titze S Foster C Oppert J-M Rutter H Oja P Sjostrom M De Bourdeaudhuij I Measuring physical activity-related environmental factors: reliability and predictive validity of the European environmental questionnaire ALPHA Int J Behav Nutr Phys Act 2010 7 48 10.1186/1479-5868-7-48 20504339 PMC2892430

[B16] Bucksch J Spittaels H Reliability and validity findings of the ALPHA environmental questionnaire in Germany J Public Health 2011 19 417 423 10.1007/s10389-011-0416-4

